# Integral kidney function assessment in pediatric patients with glycogen storage diseases

**DOI:** 10.3389/fped.2025.1543164

**Published:** 2025-04-28

**Authors:** Magali Reyes-Apodaca, Alejandra Consuelo-Sánchez, Rodrigo Vázquez-Frias, Benjamín Antonio Rodríguez-Espino, Mara Medeiros

**Affiliations:** ^1^ Faculty of Medicine, National Autonomous University of Mexico, Mexico City, Mexico; ^2^ Nephrology and Bone Mineral Metabolism Research and Diagnostic Unit, Hospital Infantil de México Federico Gómez, Mexico City, Mexico; ^3^ Department of Gastroenterology and Nutrition, Hospital Infantil de México Federico Gómez, Mexico City, Mexico; ^4^ Research Management Assistant Office, Hospital Infantil de México Federico Gómez, Mexico City, Mexico; ^5^ Research Direction Office, Hospital Infantil de México Federico Gómez, Mexico City, Mexico; ^6^ Pharmacology Department, National Autonomous University of Mexico, Mexico City, Mexico

**Keywords:** glycogen storage diseases, renal dysfunction, hyperfiltration, renal tubular acidosis, pediatric nephrology, metabolic disorders

## Abstract

**Introduction:**

Glycogen storage diseases (GSDs) are a group of hereditary metabolic disorders with variable clinical manifestations, depending on the enzyme and organ affected. Renal dysfunction, including hyperfiltration, proteinuria, and renal tubular acidosis (RTA), is a known complication, particularly in GSD types of Ia and Ib.

**Methods:**

This cross-sectional study evaluated renal function in 17 pediatric patients with different GSD types using an integral kidney assessment (IKA). The comprehensive evaluation included biochemical and urinary analyses, glomerular filtration rate calculations, and acidification tests.

**Results:**

The median age at first renal evaluation was 33 months, and nutritional management was often suboptimal at this stage. Through IKA, renal alterations were identified in 47% of the patients. Hyperfiltration was present in 40% of GSD type I patients, while lactic acidosis was noted in 30% of these cases. Two siblings with GSD XI presented with proximal RTA and Fanconi syndrome, highlighting severe tubular involvement. Distal RTA was documented in one non-adherent GSD Ia patient, underscoring the importance of metabolic control.

**Discussion:**

This study emphasizes the heterogeneity of renal manifestations among different GSD subtypes. Hyperfiltration, particularly in GSD I, may result from altered energy metabolism and compensatory mechanisms within the renal tubules. Proximal tubular damage in GSD XI reflects glycogen and monosaccharide accumulation within renal epithelial cells. Adherence to dietary and medical interventions is critical for mitigating renal complications and ensuring growth and development in GSD patients. Annual kidney evaluations are recommended for early detection of renal dysfunction, enabling timely initiation of therapeutic strategies such as alkali therapy and angiotensin-converting enzyme inhibitors.

## 1 Introduction

Glycogen storage diseases (GSDs) are a group of hereditary disorders affecting glycogen metabolism. To date, 15 types of glycogeneses have been described and are involved in different steps of the glycogen metabolic cycle. The global prevalence of GSD is estimated to range from 1 in 43,000 to 1 in 100,000 individuals. Clinical findings vary widely depending on the enzyme affected and the site where it is expressed—most commonly the liver, muscle, kidney, or heart (1–3).

In the kidney, glucose reabsorption from the glomerular filtrate in the proximal tubule depends on three key transporters: sodium–glucose cotransporter type 1 (SGLT1), sodium–glucose cotransporter type 2 (SGLT2), and glucose transporter 2 (GLUT2). SGLT1 and SGLT2 are located on the apical membrane of tubular cells, whereas GLUT2 is expressed on the basolateral membrane. SGLT2 is responsible for the majority of glucose reabsorption, whereas SGLT1 acts as a backup mechanism. GLUT2 facilitates glucose efflux from tubular cells into the bloodstream, thereby completing the reabsorption process (4).

Patients with GSD I, including GSD Ia and GSD Ib (OMIM #232200, #232220), may exhibit renal manifestations, including distal tubulopathies associated with nephrolithiasis, secondary to hypercalciuria, hypocitraturia, and hyperuricosuria. The most severe renal complication in GSD I is glomerular hyperfiltration, which may progress to microalbuminuria, significant proteinuria, glomerulosclerosis, and, ultimately, renal failure (1, 2, 4). Proximal tubulopathies have also been reported in patients with GSD I, although less frequently. These are characterized by β2-microglobulinuria, aminoaciduria, phosphaturia, hypocitraturia, and hypercalciuria (4).

In patients with Fanconi–Bickel syndrome (FBS) or GSD XI (OMIM #227810), GLUT2 dysfunction leads to abnormal glycogen accumulation in the proximal tubules, along with urinary bicarbonate loss, glucosuria, and metabolic acidosis (5). There is minimal evidence regarding renal function in other GSD types, such as GSD IIIa (OMIM #232400), which reports renal tubular acidosis (RTA) and proteinuria, and GSD IXa (OMIM #306000), which reports proximal RTA (4, 6).

This study aimed to describe the renal function of pediatric patients with glycogenesis through an integral kidney assessment (IKA).

## 2 Materials and methods

This cross-sectional study included patients with a confirmed diagnosis of GSD who attended the Hospital Infantil de México Federico Gómez. Data collected included age at clinical diagnosis, liver and metabolic biochemistry, dietary treatment, diagnosis and treatment of other renal evaluations, therapies received, and the frequency of alkali therapy if received. For the assessment of renal function, we considered two evaluations. The first was the renal assessment (FRA), which represents the first contact of the patient with the nephrology service in the hospital. The second assessment was the IKA, which was performed on all patients. The IKA included the following components: (a) blood gas analysis and anion gap calculation; (b) blood serum measurements of creatinine, uric acid, and electrolytes; (c) urine analysis including quantification of urinary electrolytes, urinary creatinine, proteinuria, glycosuria, and urinary pH, which was determined using a reagent strip using CLINITEK Multistix®; and (d) microalbuminuria, evaluated using a turbidimetric immunoassay with the HemoCue Albumin 201 equipment. Patients receiving alkali therapy (bicarbonate or citrate solution, with or without potassium) were instructed to withdraw treatment 7 days before the IKA. When the urinary pH was found to be greater than 6.5, an acidification test was performed using furosemide at 1 mg/kg/dose (7). The glomerular filtration rate (GFR) was calculated using the Revised Bedside Schwartz formula. The diagnosis of hyperfiltration was established with GFR values greater than 150 ml/min/1.73 m^2^ (7). The diagnosis of distal RTA was considered in patients presenting with hyperchloremia (>110 mmol/L), normal anion gap (<16 mEq/L), and urinary pH >5.5. Proximal RTA was diagnosed in patients with hyperchloremia, a normal anion gap, urinary pH <5.5, glucosuria, and proteinuria. Lactic acidosis was defined by the presence of low bicarbonate (<20 mmol/L), a high anion gap (>20 mEq/L), normochloremia (95–105 mmol/L), and elevated lactic acid levels (>2.0 mmol/L). Nutritional status was assessed using body mass index (BMI) and height-for-age ratio, with *z*-score cutoff points (normal ± 2 SD) established by the Centers for Disease Control and Prevention (CDC).

### 2.1 Nutritional treatment

All patients received dietary treatment that included restriction of lactose, sucrose, and fructose in conjunction with the administration of uncooked cornstarch in doses and frequency according to their type of GSD, with a fasting interval of 3–6 h maximum to maintain glucose levels above 70 mg/dl; in the case of patients with GSD III, a protein intake of at least 3 g/kg/day was ensured, while for patients with GSD type IX, the guaranteed intake was 2 g/kg/day. None of the patients received overnight infusions of glucose polymers.

### 2.2 Statistical analysis

The data obtained were processed using SPSS software for Windows, version 20.0. The Shapiro–Wilk test was performed to evaluate the distribution of variables. For qualitative variables, frequencies and proportions were estimated, while for quantitative variables, measures of central tendency and dispersion were calculated.

## 3 Results

Seventeen patients representing four different types of GSD were included; a higher proportion of girl patients was found, and the median age at diagnosis was 9 months. Thirteen patients had a first renal assessment, during which it was found that the nutritional treatment had not yet been standardized according to the type of GSD.

The median interval between the diagnosis and the first renal evaluation was 33 months; patients with GSD IIIa were evaluated later, approximately 4 years after clinical diagnosis (Table 1).

**Table 1:** General data of pediatric patients with glycogenosis.

General data	Type of GSD					
	GSD Ia *n* = 6	GSD Ib *n* = 4	GSD IIIa *n* = 3	GSD IXa *n* = 2	GSD XI *n* = 2	Total *n* = 17
Female sex, *n*	5 (83)	3 (75)	3 (100)	0	2 (100)	13 (76)
Age at clinical diagnosis of GSD (months)	11.5 (24.5)	7 (5)	7 (8)	15 (*N*D)	102.5	12 (14)
Age at the first kidney evaluation (months)	40 (58)	13 (132)	51 (28)	23.5	102.5	33
Diagnosis at the first evaluation, *n* (%)						
• RTA without classification	4 (66)	3 (75)	3 (100)	2 (100)	—	12 (70)
• Metabolic acidosis	2* (33)	—	—	—	—	2 (12)
• Fanconi syndrome	—	—	—	—	2 (100)	2 (12)
• No initial evaluation	—	1 (25)	—	—	—	1 (6)
Criteria used for initial diagnosis, *n* (%)						
High urinary pH	2 (33)	—	—	—	—	2 (12)
Low serum CO_2_	3 (50)	1 (25)	—	—	—	4 (23.5)
Urinary pH + serum CO_2_	1 (16.5)	1 (25)	2 (66.5)	—	—	4 (23.5)
Low serum HCO_3_		1 (25)		1 (50)	—	2 (12)
Unknown			1 (33.3)	1 (50)		2 (12)
Two or more criteria					2 (100)	
Alkali therapy prior to the IKA, *n* (%)	6 (100)	3 (100)	1 (33.3)	1 (50)	0	11 (64.5)
Type of alkali therapy, *n* (%)						
Citrates with K	1 (16.5)	3 (100)	—	—	2 (100)	4 (23.5)
Citrates without K	2 (33)	—	—	—		2 (12)
Sodium bicarbonate	3 (50)	—	1 (33.3)	1		5 (30)

RTA, renal tubular acidosis; CO_**2**_, carbon dioxide; HCO_**3**_, bicarbonate; ND, not determined; IKA, integral kidney assessment; K, potassium.

All age values expressed refer to estimated medians by type.

*One patient was diagnosed only with low CO_**2**_ and the other with an alkaline urinary pH.

In the FRA, 12 patients had RTA diagnoses not specified as distal or proximal, and 2 cases of metabolic acidosis were identified. The most commonly used diagnostic criteria for RTA was a combination of high urinary pH and low CO_2_ levels, followed by low CO_2_ alone as a secondary diagnostic criterion. Among patients receiving alkali therapy, a greater prescription of sodium bicarbonate was observed (Table 1).

The median age at the time of the IKA was 106 months. Short stature was observed in 11 patients; the most severe impairment was found in GSD XI, with a median *z*-score of −5.87. Four patients were classified as overweight. Based on the data collected from all 17 patients, the median values obtained for glucose, lipid profile, and uric acid indicated adequate metabolic control. However, depending on the type of GSD, variability was noted in the minimum and maximum ranges, mainly in GSD Ia, where lactate and glucose levels showed wide ranges; additionally, elevated levels of triglycerides were noted in patients with GSD Ia and GSD IIIa. At the time of the IKA, half of the patients with GSD type I were receiving allopurinol therapy (Table 2).

**Table 2:** Data of metabolic control of the patients with GSD at the time of the integral kidney assessment.

Parameter	Type of GSD					
	GSD Ia *n* = 6	GSD Ib *n* = 4	GSD III *n* = 3	GSD IXa *n* = 2	GSD XI *n* = 2	Total *n* = 17
Age, months (min–max)	127.5 (5 to 178)	74 (58 to 163)	123 (123 to 219)	105.5 (99 to 112)	99.5 (93 to 106)	106
Height, *z*-score for age (min–max)	−0.84 (−4.1 to 0.5)	−2.96 (−3.0 to −2.6)	−2.63 (−3.8 to −1.1)	−0.78 (−1.2 to −0.3)	−5.87 (−9.4 to 2.32)	−2.63
Short stature, *n* (%)	3 (50)	4 (100)	2 (66.5)	0	2 (100)	11 (65)
BMI, *z*-score (min–max)	1.3 (−1.5 to 1.6)	0.67 (0.1 to 0.8)	0.89 (0.5 to 1)	0.88 (0.51 to 1.2)	0.16 (−0.5 to 0.86)	0.72
Overweight, *n* (%)	3 (50)	0	0	1 (50)	0	4 (23.5)
Glucose (mg/dl) (min–max)	77 (45 to 122)	101 (78 to 106)	98 (82 to 115)	91 (83 to 99)	82.5 (79 to 86)	87
Triglycerides (mg/dl) (min–max)	341.5 (99 to 449)	121.5 (81 to 170)	275 (237 to 303)	80 (72 to 88)	55 (44 to 66)	170
Cholesterol-total (mg/dl) (min–max)	197 (113 to 259)	130.5 (103 to 200)	165 (165 to 225)	143 (136 to 150)	124.5 (112 to 137)	165
Uric acid (mg/dl) (min–max)	5.8* (4.6 to 6.9)	5.65* (3.6 to 7.2)	3.9 (2.2 to 5.6)	4.35 (4.3 to 4.4)	0.6 (0.3 to 0.9)	5.4
Lactate (mmol/L)	5.2 (2.8 to 7.8)	3.85 (1.3 to 4.5)	1.9 (1.1 to 1.8)	1.1 (NR)	1.1 (1 to 1.2)	2.4
HCO_3_ (mmol/L)	18.2 (14.1 to 22.8)	22.1 (20.3 to 24.5)	22.7 (18 to 24.7)	18.6 (18.1 to 19)	18.3 (17.9 to 18.7)	19.4

Min, minimum; max, maximum; BMI, body mass index; CO_2_, carbon dioxide; HCO_3_, bicarbonate.

All values expressed refer to estimated medians by type.

*Half of the patients had allopurinol treatment at the time of this determination.

In the integral kidney evaluation, two siblings with GSD XI were diagnosed with proximal RTA accompanied by Fanconi syndrome. Four patients with GSD I were identified with hyperfiltration. One 14-year-old non-adherent patient with GSD Ia presented with distal tubular acidosis and hyperfiltration and was unable to acidify the urine after receiving furosemide; therefore, citrate solution therapy was reinitiated. Four patients required sodium bicarbonate supplementation due to lactic acidosis, three of whom had GSD Ia (Table 3). After the IKA, alkali therapy was suspended in 4 of the 11 patients.

**Table 3:** Final renal diagnosis after the IKA of pediatric patients with GSD.

Renal diagnosis	Type of GSD					
	GSD Ia *n* = 6	GSD Ib *n* = 4	GSD III *n* = 3	GSD IXa *n* = 2	GSD XI *n* = 2	Total *n* = 17
No renal alteration	1 (16.5)	2 (50)	3 (100)	2 (100)	—	8 (47)
Distal renal tubular acidosis + hyperfiltration	1 (16.5)	—	—	—	—	1
Proximal renal tubular acidosis					2* (100)	2 (12)
Hyperfiltration	1 (16.5)	2 (50)	—	—		3 (17.5)
Microalbuminuria		1 (25)				1 (6)
Lactic acidosis	3	1 (25)				4

*Both cases with ATR and hyperfiltration.

## 4 Discussion

We report our experience with renal evaluation in 17 children with glucogenosis.

Renal dysfunction is a long-term complication linked to inadequate metabolic control; however, GSD XI is characterized by proximal tubulopathy and always requires alkali therapy and phosphate supplementation.

A careful renal evaluation is needed in these patients to identify those with tubulopathies, hyperfiltration, or lactic acidosis, which may benefit from targeted treatment.

In patients with GSD I, we found that hyperfiltration was the most common renal alteration in 40% of cases. Glucose-6-phosphatase (G6P) is expressed in the proximal tubular epithelium of the kidney. It is well known that the main metabolic disturbances conditioned by this enzyme make it difficult to obtain energy by glycogenolysis and gluconeogenesis, as well as the activation of futile cycles that compromise the availability of adenosine triphosphate (ATP), altering many of the ATP-dependent intracellular processes in the renal tubules. As a result of the depletion of cell energy, the GFR becomes increased (1, 2). This compensatory mechanism could explain the high frequency of renal hyperfiltration in patients with GSD; these patients have less tolerance to fasting and a higher risk of severe hypoglycemic events and lactic acidosis, mainly in the early years of life, which requires dynamic management of cornstarch or glucose dosing to maintain normal glucose, achieve metabolic control, and ensure growth and development. In some patients, achieving this balance can take several weeks after the start of nutritional treatment, and maintaining sustained metabolic control remains even more difficult. The main reason for this is that treatment adherence is complicated, and changes in growth rate, physical activity, and episodes of illness affect the patient's glucose requirements (3, 8, 9). At the time of the integral renal evaluation, at least four patients had difficulties with treatment adherence, primarily with the frequency of uncooked cornstarch and sugar restriction, which hindered effective metabolic control.

Different studies have reported that angiotensin-converting enzyme (ACE) inhibitors may be effective in reducing GFR; however, in patients with severe hypertriglyceridemia (>500 mg/dl), they have shown no effect on microalbuminuria or proteinuria. In contrast, when started early or at the first evaluation where hyperfiltration is detected, the efficacy of ACE inhibitors has been well established (3, 10).

In this study, hyperfiltration was identified in four patients with GSD I, which allowed ACEs to be initiated at the time of evaluation. In one of the patients with hyperfiltration, distal RTA was also diagnosed. It should be mentioned that these cases represented older patients who had received structured nutritional intervention at a later stage. The study by Melis et al. reinforces the importance of patient's metabolic control for the development and progression of hyperfiltration (3).

In this group of patients, those with GSD III and GSD IX did not present with kidney involvement; however, the importance of performing annual evaluations should be highlighted because of the mechanisms related to the development of kidney damage in glycogenosis. Although there are not many reports of kidney damage associated with these types, a case report by Cohen and Friedman identified two GSD III patients who presented with tubular acidosis; one of these patients developed hyperchloremic acidosis, as along with proteinuria and glycosuria, which was mainly associated with renal bicarbonate loss and, possibly, the accumulation of abnormal glycogen molecules (2, 6). Evidence of renal impairment in GSD IX is also limited; however, a recent report by Morales et al. described the presence of RTA, marked by a 50% decrease in tubular phosphate reabsorption, a decrease in urinary pH, and a low level of serum bicarbonate (11). For GSD XI, it has been reported that the proximal tubulopathy present in these patients is associated with damage to renal epithelial cells due to the accumulation of glycogen and monosaccharides (12, 13).

The use of SGLT2 inhibitors has recently been proposed as a therapeutic strategy for various forms of GSD. In murine models with GLUT2 deficiency, dapagliflozin has been shown to prevent glycogen accumulation in renal proximal tubule cells, improving renal morphology and function by promoting a metabolic shift from glycogen synthesis to glycogenolysis, thereby restoring the expression of key tubular function markers (14). Furthermore, clinical improvement has been reported in a patient with glycogen storage disease (GSD) IX treated with dapagliflozin (15).

In addition, empagliflozin has been proposed as a therapeutic alternative for GSD Ib, with evidence suggesting its potential to improve neutropenia and the associated intestinal disease (16). However, its use in the pediatric population has not yet been approved in our country. Empagliflozin may represent an innovative approach to treating GSD Ib, with the potential to significantly reduce or even eliminate the need for conventional therapies targeting neutropenia or inflammatory bowel disease. Overduin et al. recommended the use of empagliflozin in all patients with GSD Ib presenting with neutropenia at a dose of 0.3–0.4 mg/kg/day, with strict monitoring of hydration status, triglyceride levels, and urinary tract infections. They also suggested discontinuing treatment in the presence of diarrhea, vomiting, or febrile infections (16).

### 4.1 Limitations

The main limitations of our study include its single-center design and small sample size, with only 17 patients evaluated, none of whom were treated with SGLT2 inhibitors. These limitations may affect the generalizability of the findings and highlight the need for multicenter studies with a larger number of participants and prospective designs to assess long-term outcomes.

## 5 Conclusion

Metabolic control associated with therapeutic adherence in patients with GSD can affect kidney function. An integral evaluation of kidney function is essential across all types of GSD, particularly in those where renal disturbances are documented, such as GSD I, GSD III, GSD IX, and certainly FBS, to identify early emerging alterations and make appropriate therapeutic interventions.

## Data Availability

The raw data supporting the conclusions of this article will be made available by the authors without undue reservation.
